# A Focal Pontine Infarct Presenting as Unilateral Facial Nerve Paralysis

**DOI:** 10.7759/cureus.10646

**Published:** 2020-09-25

**Authors:** Kelsey Burson, Joshua Mastenbrook, Kyle Van Dommelen, Mauli Shah, Laura D Bauler

**Affiliations:** 1 Department of Emergency Medicine, Western Michigan University Homer Stryker MD School of Medicine, Kalamazoo, USA; 2 Department of Biomedical Sciences, Western Michigan University Homer Stryker MD School of Medicine, Kalamazoo, USA

**Keywords:** bell’s palsy, pontine infarct, stroke, facial paralysis

## Abstract

Brainstem infarction typically presents with vague symptoms, including headache, nausea, vomiting, and vertigo. Rarely do patients present with complete unilateral facial paralysis, mimicking Bell’s palsy. Here we report the case of a 40-year-old woman who presented to the emergency department with intractable nausea, vomiting, and vertigo upon waking along with left-sided upper and lower extremity numbness and right-sided facial paralysis. Her atypical presentation of unilateral facial nerve paralysis in the context of nausea, vomiting, and vertigo prompted neurological studies, which were significant for a small punctate infarct in the pons involving the right facial colliculus. ​History, physical examination, and clinical suspicion are important to prevent anchoring bias. Physicians rely on history and physical examination to help distinguish true Bell’s palsy from other causes of facial nerve paralysis. Stroke and other clinically emergent etiologies should be considered high on the differential diagnosis when patients have neurological signs and symptoms in addition to facial nerve palsy.

## Introduction

Brainstem strokes account for 10% of ischemic strokes, and are most commonly found in the pons. The symptoms are often vague, including dizziness, visual-field disturbances, nausea, and vomiting [1]. Rapid diagnosis and treatment is essential for a full recovery. Here we describe a 40-year-old female with minimal stroke risk factors who had a focal pontine infarction that manifested similar to Bell’s palsy with complete unilateral facial paralysis. Many clinically emergent etiologies, such as stroke, can present with Bell’s palsy-like symptoms and should be excluded before considering a diagnosis of Bell’s palsy, even in patients who lack common risk factors.

## Case presentation

A previously healthy 40-year-old female presented to the emergency department with symptoms of movement-exacerbated room-spinning vertigo, nausea, and vomiting upon waking in the morning. She also reported noticing numbness of her left upper and lower extremities, but denied any focal weakness, vision or speech changes, headache, trauma, rash, or illicit drug use. Her only current medication was an oral contraceptive, norgestimate-ethinyl estradiol 0.25 mg to 35 mcg. The patient was alert and oriented, afebrile, with a heart rate of 81 beats per minute, and a blood pressure of 141/71 mmHg. It was immediately noted on physical exam that the patient had complete right-sided facial paralysis, instinctively raising suspicion for Bell’s palsy. She did not display any dysarthria, dysphagia, or diplopia. With the exception of complete cranial nerve VII palsy, detailed bedside neurological examination did not reveal any other obvious focus neural deficits. Horizontal bidirectional nystagmus was present on end lateral gaze, but no vertical nystagmus was noted. Cerebellar function was evaluated with finger-to-nose and heel-along-shin testing, both of which did not display any abnormalities. She was not ambulated in the emergency department. Examination did not reveal any objective sensory or motor deficits in the bilateral upper or lower extremities. There was no nuchal rigidity or rash present. The patient was initially treated with normal saline (2 L), ondansetron (4 mg), and promethazine (12.5 mg), which helped minimize her symptoms. Initial laboratory tests were significant for leukocytosis, lactic acidosis, and minimally elevated cholesterol levels (Table 1).

**Table 1:** Laboratory Values * Indicates significant results

Laboratory Values	Result	Normal Range
White blood cell count	18.2 x 10^9^ cells/L*	4.0-11.0 x 10^9^ cells/L
Sodium	135 mmol/L	135-145 mmol/L
Potassium	3 mmol/L	3.5-5.3 mol/L
Glucose	172 mg/dL*	70-99 mg/dL
Lactic acid	8.4 mmol/L*	0.7-2.5 mmol/L
Ethanol	<0.01 g/dL	<0.01 g/dL
Urine drug screen 6	Negative	Negative
Urine pregnancy test	Negative	Negative
Total cholesterol	227 mg/dL*	125-200 mg/dL
Low-density lipoprotein	151 mg/dL*	<100 mg/dL
High-density lipoprotein	54 mg/dL	>50 mg/dL
Hypercoagulable workup	Result	Normal range
Anticardiolipin antibody	Negative	Negative
Factor VIII screen	Negative	Negative
Antithrombin III activity	Negative	Negative
Factor V Leiden	Negative	Negative
Factor II genotype	Negative	Negative
Lupus anticoagulation screen	Negative	Negative
Protein C and S studies	Negative	Negative
Prothrombin time/international normalized ratio	11.4 seconds/1	10-12.5 seconds/less than 2
Activated partial thromboplastin time	23 seconds	25-37 seconds

**Figure 1:**
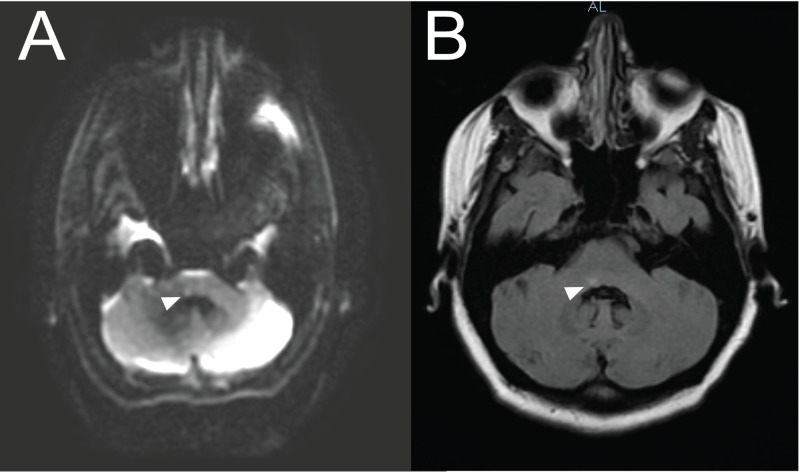
Brain MRI (A) Initial MRI (with contrast) showing increased focus on the diffusion-weighted imaging series in the dorsal aspect of the right pons at the floor of the fourth ventricle corresponding to the right facial colliculus, indicated by the arrowhead. (B) Second MRI performed on day 2 of admission showing an increased focus in the pons, indicated by the arrowhead.

An electrocardiogram did not reveal an arrhythmia, specifically atrial fibrillation, a known risk factor for embolic stroke. Due to concern for other neurological etiologies given the patient’s sudden onset of vertiginous symptoms and subjective left extremity paresthesias, CT of the head without contrast was performed. CT results did not reveal any space occupying lesion, acute hemorrhage, or infarct. Based on the patient’s present illness, vital signs, physical exam findings, and CT results, there was lower suspicion for a tumor, hemorrhage, or acute ischemic infarct causing the complete facial nerve paralysis; however, the latter could not be completely ruled out by CT alone. Although the patient was clinically stable, she was admitted to the hospital due to persistent vertigo and unexplained complete right facial paralysis. MRI of the brain with and without contrast was performed shortly after admission. Results were read by a board-certified neuroradiologist and were initially reported as negative for any abnormal pathology. However, within the hour, a discussion between the neurologist and neuroradiologist stimulated re-evaluation of the imaging, elucidating a discrete, punctate focus of increased signal in the region of the right facial colliculus, suggestive of ischemia (Figure 1A). This was noted to have increased in size when imaged on day 2 (Figure 1B). Further investigation into the potential cause of the ischemic injury with magnetic resonance angiography of the head and neck, and a transesophageal echocardiogram, was unrevealing. The patient had no known personal or family history of a hypercoagulable disorder, and her hypercoagulability panel results were within normal limits (Table 1). After a four-day admission, the patient was discharged home on daily aspirin (325 mg) and atorvastatin (40 mg), encouraged to discontinue her oral contraceptive, and was referred for outpatient follow-up with a speech therapist, physical therapist, and a local stroke clinic.

## Discussion

Complete right-sided facial paralysis in the setting of a stroke is uncommon [2,3]. Bell’s palsy, the most common etiology of facial nerve paralysis, is a temporary and self-limiting unilateral facial paralysis affecting approximately 40,000 individuals annually in the United States [3-6]. Symptom onset is acute and most common in individuals between the ages of 15 and 45 years [5]. Strokes with facial involvement on the other hand typically affect a unilateral quadrant of the face rather than the unilateral upper and lower quadrants of the face seen in Bell’s palsy. Common risk factors for stroke include advanced age, hypertension, smoking, hyperlipidemia, diabetes, and cardiovascular disease [7]. The 40-year old patient had none of these risk factors known upon her initial presentation to the emergency department. Despite further investigation, no evidence of a thromboembolic event or hypercoagulable blood disorder was identified. The potential risk factors ultimately identified in the patient were the presence of mild hypercholesterolemia and use of an oral contraceptive. Although rare, low-estrogen oral contraceptives increase the risk of ischemic stroke by twofold, from 4.4 to 8.5 per 100,000 [8].

Our patient had a focal punctate ischemic insult in the right facial colliculus located in the pons, which resulted in the complete loss of function of the ipsilateral facial nerve, mimicking Bell’s palsy. Loss of function of the ipsilateral facial nerve can be visually assessed by an inability to wrinkle the forehead, inability to completely or forcefully close the eyelids, and evidence of unilateral facial droop upon smiling; however, these signs will also be seen in patients with Bell's palsy. For patients with persistent vertigo, as was seen in our patient, the HINTS test, a diagnostic tool, can help differentiate between a central and peripheral etiology of vertigo. HINTS is an acronym for head impulse, nystagmus, and test of skew [9]. HINTS tests positive for a normal head impulse, bidirectional nystagmus, or abnormal test of skew are suggestive of a central etiology. For our patient with complete hemi-facial paralysis, symptoms of persistent vertigo (positive for bidirectional nystagmus), nausea, vomiting, and subjective unilateral paresthesias supported concern for a central etiology rather than a peripheral cause, prompting further imaging.

Strokes of the motor cortex typically present with lower facial involvement given that the forehead receives motor innervation from both hemispheres of the cerebral cortex. However, as this case demonstrates, ischemic strokes may present with complete hemi-facial paralysis mimicking Bell’s palsy depending upon the location of the infarction [3]. Anatomically, the anterior aspect of the facial motor nucleus receives corticobulbar fibers from the contralateral cortex and supplies motor function to the lower half of the ipsilateral face. The posterior aspect of the facial motor nucleus receives corticobulbar fibers from bilateral cortices and supplies the ipsilateral forehead [10]. As a result of these aforementioned neuronal pathways, one can understand the classic teaching that a cortical stroke should only have lower facial involvement with forehead sparing. However, should a stroke insult the facial colliculus, physical examination may reveal complete hemi-facial paralysis mimicking Bell’s palsy. The majority of these vascular insults are lacunar infarcts involving the small perforating arterioles from the basilar artery and other posterior circulation vessels [2,11]. A study from the United Kingdom extrapolated that upwards of 9,000 pontine strokes may be diagnosed as Bell’s palsy every year [3]. Vascular pontine lesions remain rare, representing 7% of all ischemic strokes, and 1% of facial nerve palsies [2,3].

One similar case was identified in the literature, describing a 47-year-old male with a cranial nerve VII palsy due to a pontine infarct. However, unlike our patient, he had a history of hypertension, a well-recognized risk factor for stroke. It is important to note some key similarities between these cases. Both patients did present with symptoms of nausea and vomiting. In addition, there was difficulty in identifying the infarction on MRI given its location and size [2], as well as the confounding presentation of complete hemi-facial paralysis mimicking Bell’s palsy.

## Conclusions

Rapid identification of stroke is crucial to avoid associated morbidity and mortality. This case demonstrates the importance of a complete neurological examination in any patient with a cranial nerve VII palsy. For our patient, despite minimal risk factors for ischemic stroke (mild hypercholesterolemia and oral contraceptive use), the additional symptoms of unilateral paresthesias, vertigo, nausea, and vomiting supported a central etiology over Bell's palsy. Heightened awareness for a subtle pontine stroke in patients with unilateral facial paralysis is paramount given that confirmation of stroke can be difficult even with MRI, as demonstrated by this case.
